# Charakteristika und Outcome von 70 beatmeten COVID-19-Patienten: Bilanz nach der ersten Welle an einem universitären Zentrum

**DOI:** 10.1007/s00101-020-00906-3

**Published:** 2020-12-28

**Authors:** Ines Schroeder, Christina Scharf, Michael Zoller, Dietmar Wassilowsky, Sandra Frank, Stephanie-Susanne Stecher, Joachim Stemmler, Nikolaus Kneidinger, Sven Peterß, Bernhard Zwißler, Michael Irlbeck

**Affiliations:** 1 Klinik für Anästhesiologie, LMU Klinikum, Marchioninistr. 15, 81377 München, Deutschland; 2 Medizinische Klinik und Poliklinik II, LMU Klinikum, Marchioninistr. 15, 81377 München, Deutschland; 3 Medizinische Klinik und Poliklinik III, LMU Klinikum, Marchioninistr. 15, 81377 München, Deutschland; 4 Medizinische Klinik und Poliklinik V, Deutsches Zentrum für Lungenforschung (DZL), LMU Klinikum, Marchioninistr. 15, 81377 München, Deutschland; 5 Herzchirurgische Klinik und Poliklinik, LMU Klinikum, Marchioninistr. 15, 81377 München, Deutschland

**Keywords:** ECMO, Akutes Lungenversagen, Letalität, Intensivtherapie, ARDS-Zentrum, ECMO, Acute respiratory distress syndrome, Mortality, Intensive care therapy, ARDS specialist center

## Abstract

**Hintergrund:**

Eine aktuelle, deutschlandweite Datenerhebung zeigte bei beatmeten Patienten mit COVID-19 eine Letalität von über 50 %. Auch am LMU Klinikum wurde eine große Anzahl an Patienten mit COVID-19 mit teils erheblicher Erkrankungsschwere intensivmedizinisch behandelt.

**Fragestellung:**

Die Daten der am LMU-Klinikum behandelten COVID-19-Patienten wurden systematisch ausgewertet und mit den deutschlandweiten Daten verglichen.

**Methodik:**

Für die vorliegende Studie wurden die Daten aller Patienten, die bis zum 31.07.2020 am LMU-Klinikum aufgrund von COVID-19 invasiv und nichtinvasiv beatmet wurden und deren Krankenhausaufenthalt zum Zeitpunkt der Auswertung bereits abgeschlossen war, analysiert und mittels deskriptiver Statistik aufgearbeitet.

**Ergebnisse:**

Insgesamt wurden 70 kritisch kranke, beatmete Patienten (SAPS-II-Median: 62 Punkte) analysiert (Altersmedian: 66 Jahre, 81 % männlich). Über 90 % wurden wegen eines akuten Lungenversagens (ARDS) unterschiedlicher Schweregrade behandelt. Eine Therapie mittels extrakorporaler Membranoxygenierung (ECMO) war bei 10 % erforderlich. Die Übernahme von externen Kliniken im Rahmen einer ARDS/ECMO-Anfrage erfolgte bei 27,1 % der Patienten. Häufig eingesetzte immunmodulatorische Therapien waren die Behandlung mit Cytosorb® (18,6 %) und die prolongierte Gabe von Methylprednisolon (25,7 %). Die krankenhausinterne Letalität betrug 28,6 %.

**Fazit:**

Trotz erheblicher Erkrankungsschwere lag die Letalität bei beatmeten COVID-19-Intensivpatienten im LMU-Kollektiv deutlich unter der deutschlandweit erhobenen Letalität. Ein möglicher Faktor ist die Behandlung in einem Zentrum für ARDS.

## Abstract (en)

**Background:**

In a pandemic situation the overall mortality rate is of considerable interest; however, these data must always be seen in relation to the given healthcare system and the availability of local level of care. A recently published German data evaluation of more than 10,000 COVID-19 patients treated in 920 hospitals showed a high mortality rate of 22% in hospitalized patients and of more than 50% in patients requiring invasive ventilation. Because of the high infection rates in Bavaria, a large number of COVID-19 patients with considerable severity of disease were treated at the intensive care units of the LMU hospital. The LMU hospital is a university hospital and a specialized referral center for the treatment of patients with acute respiratory distress syndrome (ARDS).

**Objective:**

Data of LMU intensive care unit (ICU) patients were systematically evaluated and compared with the recently published German data.

**Methods:**

Data of all COVID-19 patients with invasive and noninvasive ventilation and with completed admission at the ICU of the LMU hospital until 31 July 2020 were collected. Data were processed using descriptive statistics.

**Results:**

In total 70 critically ill patients were included in the data evaluation. The median SAPS II on admission to the ICU was 62 points. The median age was 66 years and 81% of the patients were male. More than 90% were diagnosed with ARDS and received invasive ventilation. Treatment with extracorporeal membrane oxygenation (ECMO) was necessary in 10% of the patients. The median duration of ventilation was 16 days, whereby 34.3% of patients required a tracheostomy. Of the patients 27.1% were transferred to the LMU hospital from external hospitals with reference to our ARDS/ECMO program. Patients from external hospitals had ARDS of higher severity than the total study population. In total, nine different substances were used for virus-specific treatment of COVID-19. The most frequently used substances were hydroxychloroquine and azithromycin. Immunomodulatory treatment, such as Cytosorb® (18.6%) and methylprednisolone (25.7%) were also frequently used. The overall in-hospital mortality rate of ICU patients requiring ventilation was 28.6%. The mortality rates of patients from external hospitals, patients with renal replacement therapy and patients with ECMO therapy were 47.4%, 56.7% and 85.7%, respectively.

**Conclusion:**

The mortality rate in the ventilated COVID-19 intensive care patients was considerably different from the general rate in Germany. The data showed that treatment in an ARDS referral center could result in a lower mortality rate. Low-dose administration of steroids may be another factor to improve patient outcome in a preselected patient population. In the authors’ opinion, critically ill COVID-19 patients should be treated in an ARDS center provided that sufficient resources are available.

In einem pandemischen Geschehen wie aktuell bedingt durch COVID-19 sind Letalitätsdaten von großem Interesse, wobei diese immer vor dem Hintergrund der Ressourcen des jeweiligen Gesundheitssystems gesehen werden müssen.

Unlängst wurde eine deutschlandweite Studie von über 10.000 COVID-19-Patienten aus 920 Krankenhäusern aller Versorgungsstufen veröffentlicht [1]. Dabei zeigten sich Letalitätsraten von 22 % der hospitalisierten Patienten und über 50 % der beatmeten Intensivpatienten. Diese Ergebnisse wurden mit Daten aus einem universitären Zentrum für akutes Lungenversagen (ARDS) verglichen.

## Hintergrund

Trotz intensiver Forschung ist immer noch zu wenig über COVID-19 bekannt. Das Infektionsgeschehen ist zu jung für abschließende demografische Bewertungen, da sich international viele Patienten noch immer in Behandlung befinden. Zudem müssen v. a. Parameter wie die Letalität in einem pandemischen Geschehen immer auch vor dem Hintergrund der Ressource des jeweiligen Gesundheitssystems gesehen werden. In Deutschland blieb der Kollaps des Gesundheitssystems bisher aus. Selbst zum bisherigen Höhepunkt der Pandemie Ende März standen freie Intensivbetten zur Verfügung. Die erste Krankheitswelle ist abgeflaut, sodass abgeschlossene Behandlungsverläufe analysiert werden können. Letalitätsdaten aus Deutschland sind daher von besonderem Interesse.

Unlängst wurde eine deutschlandweite Studie veröffentlicht, die die Daten von über 10.000 COVID-19-Patienten aus 920 Krankenhäusern aller Versorgungsstufen analysiert und damit erstmals bundesweite und bevölkerungsrepräsentative Ergebnisse vorgestellt hat [1]. Insgesamt verstarben in Deutschland etwa 22 % der hospitalisierten und über 50 % der beatmeten Intensivpatienten.

Aufgrund der hohen Infektionszahlen in Bayern wurde am LMU-Klinikum eine große Anzahl an COVID-19-Patienten behandelt. Als Zentrum für akutes Lungenversagen (ARDS) und Behandlung mit extrakorporaler Membranoxygenierung (ECMO) erfolgte die Zuverlegung einiger besonders schwer betroffener Patienten aus externen Krankenhäusern.

In der vorliegenden Studie werden diese Patienten analysiert und ins Verhältnis zu den deutschlandweit, aber auch international erhobenen Daten gesetzt.

## Methodik

### Datenquelle

Die vorgestellte Studie ist eine monozentrische, retrospektive Datenerhebung aller Patienten, die am LMU-Klinikum bis zum 31.07.2020 intensivmedizinisch aufgrund von COVID-19 behandelt wurden. Die ansässige Ethikkommission stimmte der Datenerhebung und -auswertung zu (CORKUM, WHO trial ID DRKS00021225).

### Studienpopulation

Es wurden alle Patienten eingeschlossen, die im oben genannten Zeitraum mit der Diagnose COVID-19 (SARS-CoV-2-Infektion bestätigt durch PCR) invasiv und nichtinvasiv beatmet wurden, und deren Krankenhausaufenthalt bereits abgeschlossen war (Patient wurde bereits aus dem Krankenhaus entlassen oder ist verstorben).

### Datenerhebung

Alle studienrelevanten Parameter wurden der Patientenakte entnommen. Diese beinhaltete demografische Parameter, behandlungsspezifische Parameter und Parameter zu Beatmung, Organersatz- und ECMO-Therapie. Der Schweregrad des ARDS wurde nach der Berliner Definition klassifiziert [2]. Der SAPS II wurde als Kennzahl für die Krankheitsschwere bei Aufnahme evaluiert [3]. Pulmonale Ko- oder Superinfektionen wurden als relevanter Keimnachweis (Keimnachweis führte zu Therapie und Diagnose) aus respiratorischem Material definiert. Das akute Leberversagen wurde definiert als Gesamtbilirubin >10 mg/dl, und/oder Spontan-INR >1,5 und/oder Diagnose durch behandelnden Arzt. Bei den Todesfällen wurde untersucht, ob die Patienten „an“ oder „mit“ COVID-19 verstorben sind. Als „verstorben an“ wurden die Patienten definiert, bei denen die Infektion und deren Folgen unmittelbar zum Tode geführt haben (ohne Infektion wäre der Patient nicht zum gegebenen Zeitpunkt gestorben). Als „verstorben mit“ wurden die Patienten definiert, bei denen andere Faktoren ursächlich zum Tode geführt haben und der Infektion eine untergeordnete Rolle zukommt (ohne Infektion wäre der Patient zum gegebenen Zeitpunkt dennoch verstorben).

### Statistische Auswertung

Stetige Variablen wurden als Median mit „interquartile range“ (IQR) angegeben, während bei kategorischen Variablen die Anzahl mit Prozent angegeben wurde. Mithilfe deskriptiver Statistik wurden verschiedene Gruppen charakterisiert.

## Ergebnisse

### Organisation der Intensivstationen

Das LMU-Klinikum ist ein Hochschulklinikum der III. Versorgungsstufe. Während der COVID-19-Pandemie wurden alle Intensivbetten zentral koordiniert. In der Hochphase der Pandemie wurden 4 Intensivstationen ausschließlich für die Versorgung von COVID-19-Patienten genutzt. Die Patienten wurden anhand eines interdisziplinär erstellten Therapiestandards behandelt, der regelmäßig an neue wissenschaftliche Erkenntnisse angepasst wurde. Dieser enthielt auch eine Empfehlung zur strategischen Herangehensweise zu Atemwegssicherung und Beatmung bei COVID-19-Patienten: Vor der Intubation erfolgte eine engmaschige Überwachung auf Zeichen der erhöhten Atemarbeit mit drohender respiratorischer Erschöpfung anhand von objektivierbaren Kriterien und durch die klinische Einschätzung eines intensivmedizinisch erfahrenen Arztes. Bei drohender respiratorischer Erschöpfung (Atemfrequenz >35/min, p_a_CO_2_ <30 mm Hg, Lactat >2 mmol/l, p_a_O_2_ <60 mm Hg unter max. 8 l O_2_-Insufflation über eine Maske mit einem Reservoirbeutel) wurden die Patienten durch ein erfahrenes Anästhesieteam intubiert und auf die Intensivstation verlegt. Eine primäre NIV-Therapie erfolgte nur in speziellen Ausnahmesituationen (z. B. bei Patienten mit einer „do-not-intubate order“). Die intensivmedizinischen Behandlungskapazitäten waren zu keinem Zeitpunkt ausgeschöpft.

### Demografische Daten der COVID-19-Intensivpatienten

Insgesamt 70 beatmete Patienten mit gesicherter COVID-19-Diagnose wurden in die Datenauswertung eingeschlossen und werden im Folgenden als Studienpopulation angesehen. Das mediane Alter lag bei 66 Jahren, und 81 % der Patienten waren männlich. Der SAPS II 24 h nach Aufnahme betrug im Median 62 Punkte. Viele Patienten hatten relevante Vorerkrankungen wie arterielle Hypertonie (64,3 %) oder Diabetes mellitus (31,4 %). Nur 5 Patienten (7,1 %) wiesen keine Komorbidität auf. Fast alle Patienten (91,4 %) erfüllten die ARDS-Kriterien nach der Berliner Definition.

27,1 % aller Patienten wurden im Rahmen einer ARDS/ECMO-Anfrage von externen Kliniken übernommen. Im Median wurden die Patienten 18 Tage intensivmedizinisch versorgt.

Eine genaue Übersicht über Patientencharakteristika, Komorbiditäten und den Schweregrad des ARDS gibt Tab. 1.

**Table 1:** 

		Alle Patienten	>14 Tage invasive Beatmung	ECMO-Therapie	Verstorbene Patienten	Patienten aus externen Kliniken
*Patientencharakteristika*						
Anzahl (%)		70 (100)	36 (51,4)	7 (10)	20 (28,6)	19 (27,1)
Alter – Median (IQR)		66 (57, 74)	69 (60, 73)	66 (62, 71)	69 (64, 79)	64 (54, 72)
Männlich (%)		57 (81,4)	28 (77,8)	5 (71,4)	18 (90)	17 (89,5)
SAPS II bei Aufnahme ICU – Median (IQR)		62 (48, 69)	65 (58, 70)	70 (59, 81)	68 (62, 81)	62 (54, 69)
*Komorbiditäten*						
Arterielle Hypertonie (%)		45 (64,3)	25 (69,4)	5 (71,4)	13 (65)	12 (63,2)
Koronare Herzerkrankung (%)		21 (30,0)	12 (33,3)	2 (28,6)	11 (55)	10 (52,6)
Pulmonale Vorerkrankung (%)		19 (27,1)	10 (27,8)	2 (28,6)	6 (30)	5 (26,3)
Nikotinabusus >20 pack years (%)		11 (15,7)	7 (19,4)	2 (28,6)	2 (10)	4 (21,1)
Diabetes mellitus (%)		22 (31,4)	13 (36,1)	3 (42,9)	11 (55)	9 (47,4)
BMI >30 m^2^/kg (%)		20 (28,6)	14 (38,9)	3 (42,9)	5 (25)	7 (36,8)
Chronische Niereninsuffizienz (%)		10 (14,3)	6 (16,7)	1 (14,3)	5 (25)	3 (15,8)
Aktuelle hämatoonkologische Erkrankung (%)		8 (11,4)	5 (13,9)	0 (0,0)	3 (15,0)	1 (5,3)
*ARDS*						
Diagnose ARDS (%)		64 (91,4)	33 (91,7)	7 (100)	19 (95)	18 (94,7)
Mildes ARDS (%)		11 (17,2)	3 (9,1)	0 (0)	1 (5,3)	0 (0,0)
Moderates ARDS (%)		23 (35,9)	8 (24,2)	1 (14,3)	5 (26,3)	6 (33,3)
Schweres ARDS (%)		30 (46,9)	22 (66,7)	6 (85,7)	13 (68,4)	12 (66,7)
*Intensivmedizinische Behandlung*						
Nichtinvasive Beatmung (%)		4 (5,7)	0 (0)	0 (0)	0 (0)	0 (0,0)
Invasive Beatmung	Anzahl (%)	66 (94,3)	36 (100)	7 (100)	20 (100)	19 (100,0)
	Dauer (Tage) – Median (IQR)	16 (8, 29)	27 (20, 43)	22 (17, 27)	21 (13, 28)	23 (14, 30)
Aufnahme aus externem Krankenhaus (%)		19 (27,1)	14 (38,9)	4 (57,1)	9 (45)	19 (100,0)
Tage bis Verlegung oder Tod – Median (IQR)		18 (10, 28)	27 (20, 50)	21 (16, 22)	14 (21, 31)	17 (11, 24)
Tracheotomie (%)		24 (34,3)	23 (63,9)	2 (28,6)	7 (35)	9 (47,4)
Bauchlagerung (%)		31 (44,3)	20 (55,6)	7 (100)	11 (55)	16 (84,2)
Nierenersatzverfahren wie Hämodialyse/-filtration (%)		30 (42,9)	23 (63,9)	7 (100)	17 (85)	10 (52,6)
*Antivirale Therapie*						
Ritonavir + Lopinavir (%)		4 (5,7)	2 (5,6)	0 (0)	1 (5)	0 (0,0)
Remdesivir (%)		1 (1,4)	0 (0)	0 (0)	0 (0)	0 (0,0)
Favipiravir (%)		1(1,4)	1 (2,8)	1 (14,3)	1 (5)	1 (5,3)
Hydroxychloroquin (%)		36 (51,4)	18 (50,0)	4 (57,1)	11 (55)	11 (57,9)
Azithromycin (%)		50 (71,4)	26 (72,2)	5 (71,4)	15 (75)	11 (57,9)
Rekonvaleszentenplasma (%)		3 (4,3)	3 (8,3)	1 (14,3)	1 (5)	1 (5,3)
Interferon‑β (%)		1 (1,4)	1 (2,8)	0 (0)	1 (5)	0 (0,0)
*Immunmodulatorische Therapie*						
Methylprednisolon (%)		18 (25,7)	11 (30,6)	3 (42,9)	7 (35)	5 (26,3)
Tocilizumab (%)		6 (8,6)	4 (11,1)	3 (42,9)	3 (15)	2 (10,5)
Cytosorb® (%)		13 (18,6)	10 (27,8)	5 (71,4)	10 (50)	4 (21,1)
*Komplikationen im Verlauf*						
Akutes Leberversagen (%)		14 (20)	12 (33,3)	5 (71,4)	12 (60)	8 (42,1)
Thrombembolisches Ereignis (%)		15 (21,4)	9 (25)	1 (14,3)	6 (30)	7 (36,8)
*Pulmonale Ko‑/Superinfektionen*						
Bakteriell (%)		12 (17,1)	9 (25)	2 (28,6)	4 (20)	4 (21,1)
Viral (%)		5 (7,1)	4 (11,1)	0 (0)	2 (10)	2 (10,5)
Fungal (%) (*Aspergillus-Spezies*)		5 (7,1)	4 (11,1)	1 (14,3)	4 (20)	2 (10,5)
*Letalität*						
Verstorben (%)		20 (28,6)	14 (38,9)	6 (85,7)	20 (100)	9 (47,4)
Verstorben an COVID-19 (%)		17 (85)	13 (92,9)	6 (100)	17 (85)	9 (100,0)
Verstorben mit COVID-19 (%)		3 (15)	1 (7,1)	0 (0)	3 (15)	0 (0,0)

*SAPS* „simplified acute physiology score“, *BMI* Body-Mass-Index, *ARDS* „acute respiratory distress syndrome“, *IQR* „interquartile range“, *COVID* „coronavirus disease“

### Beatmung und ECMO-Therapie

94,3 % der Patienten wurden invasiv beatmet. Die Dauer der Beatmung betrug im Median 16 Tage. Bei 7 Patienten (10 %) wurde die Indikation zum Lungenersatz mittels venovenöser ECMO gestellt.

Die von externen Krankenhäusern übernommenen Patienten hatten ARDS höherer Schweregrade (66,7 % mit schwerem ARDS) als das Gesamtkollektiv. Fast alle (95 %) der von extern übernommenen Patienten waren zum Zeitpunkt der Übernahme bereits invasiv beatmet.

Detaillierte Informationen zur intensivmedizinischen Behandlung finden sich in Tab. 1 („intensivmedizinische Behandlung“).

### Medikamentöse Therapie der COVID-19-Infektion

Insgesamt kamen im Sinne einer virusspezifischen Therapie 9 verschiedene Substanzen zum Einsatz. Häufig verwendete Substanzen waren Hydroxychloroquin (51,4 %) und Azithromycin (71,4 %). Im zeitlichen Verlauf der Pandemie wurden zunehmend weniger antivirale Medikamente verwendet. Im Sinne einer immunmodulatorischen Therapie wurde bei 18,6 % der Patienten der Zytokinabsorber Cytosorb® eingesetzt. Bei 25,7 % der Patienten wurde Methylprednisolon verabreicht.

Detaillierte Informationen zur Häufigkeit des Einsatzes der einzelnen Substanzen finden sich in Tab. 1 („antivirale und immunmodulatorische Therapie“).

### Komplikationen im Verlauf

Relevante Komplikationen im Verlauf der Infektion waren ein akutes, dialysepflichtiges Nierenversagen (42,9 %), thrombembolische Ereignisse (21,4 %) und ein akutes Leberversagen (20,0 %).

Bei 18 Patienten (25,7 %) wurden während des intensivmedizinischen Verlaufs insgesamt 22 relevante Keime aus respiratorischem Material nachgewiesen. Insbesondere der Nachweis einer invasiven pulmonalen Aspergillose war mit einer Letalität von 80 % assoziiert.

Weitere Details hierzu befinden sich in Tab. 1 („Komplikationen im Verlauf und pulmonale Ko‑/Superinfektionen“).

### Letalitätsdaten

Die Letalität im gesamten Patientenkollektiv betrug 28,6 %, wobei 85 % der Patienten „an“ und 15 % „mit“ COVID-19 gestorben sind. Mit 47,4 % lag die Letalität der Patienten, die von externen Krankenhäusern übernommen wurden, deutlich über der Gesamtletalität der Kohorte. Die Letalitätsraten bei speziellen Patientenkollektiven und Diagnosen zeigt Tab. 2. Hervorzuheben ist, dass mehr als die Hälfte der Patienten mit schwerem ARDS (Horowitz-Index < 100 mmHg) überlebt haben, die Beatmungsdauer keinen Einfluss auf die Letalität hatte und die Letalitätsrate bei Patienten mit akutem Nierenversagen und der Notwendigkeit zur Nierenersatztherapie 56,7 % betrug.

**Table 2:** 

	Verstorben *n* (%)	Überlebend *n* (%)
*Alter*		
<60 Jahre	2 (9,5)	19 (90,5)
60–69 Jahre	8 (40)	12 (60)
70–79 Jahre	5 (23,8)	16 (76,2)
>79 Jahre	5 (62,5)	3 (37,5)
*ARDS*		
Mildes ARDS (%)	1 (9,1)	10 (90,9)
Moderates ARDS (%)	5 (21,7)	18 (78,3)
Schweres ARDS (%)	13 (43,3)	17 (56,7)
ARDS mit ECMO (%)	6 (85,7)	1 (14,3)
*Beatmung*		
Nichtinvasive Beatmung (%)	0	4 (100)
<14 Tage invasive Beatmung (%)	14 (41,2)	20 (58,8)
>14 Tage invasive Beatmung (%)	14 (38,9)	22 (61,1)
Tracheotomie (%)	7 (29,2)	17 (70,8)
Bauchlagerung (%)	12 (38,7)	19 (61,3)
*Komplikationen im Verlauf*		
Thrombembolisches Ereignis (%)	6 (40,0)	9 (60,0)
Hämodialyse bei akutem Nierenversagen (%)	17 (56,7)	13 (43,3)
Akutes Leberversagen (%)	12 (85,7)	2 (14,3)
*Patienten*		
Alle Patienten (%)	20 (28,6)	50 (71,4)
Primär an LMU stationär aufgenommen (%)	11 (21,6)	40 (78,4)
Von extern übernommen (%)	9 (47,4)	10 (52,6)

*ARDS* „acute respiratory distress syndrome“, *ECMO* extrakorporale Membranoxygenierung

**Figure 1:**
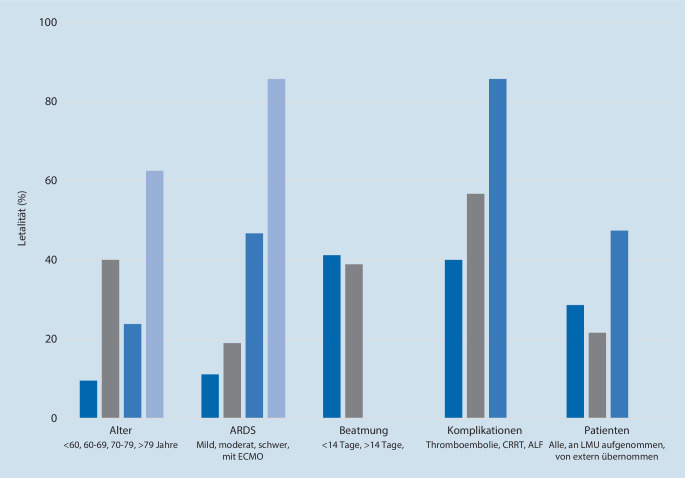

In Abb. 1 wird die Letalität, abhängig von Alter, Schweregrad des ARDS, Beatmung, Komplikationen und Aufnahmeklinik, dargestellt.

## Diskussion

Insgesamt variiert die international beschriebene Letalität schwer erkrankter COVID-19-Patienten stark, liegt aber mit Werten zwischen 26 %[4], 32 % [5], 38 % [6], 78 % [7] bis 88 % [8] überwiegend über der von uns beschriebenen Letalität.

Ein direkter Vergleich ist jedoch schwierig:

Viele Daten wurden zu einem Zeitpunkt veröffentlicht, zu dem die Krankenhausbehandlung bei einem Großteil der Patienten noch nicht abgeschlossen war [4–8]. So berichten Richardson et al. in ihrem großen New Yorker Kollektiv eine Letalität von 88 % bei invasiv beatmeten Patienten mit abgeschlossenem Behandlungsverlauf. Über 70 % der Patienten befanden sich jedoch zum Zeitpunkt der Veröffentlichung noch in stationärer Behandlung. Die Entwicklung der Letalität nach Abschluss der stationären Behandlung ist somit spekulativ.

Des Weiteren unterstellen wir einen Zusammenhang zwischen einer Überforderung der Gesundheitssysteme, die in vielen Ländern eingetreten ist, und steigenden Letalitätszahlen.

Der deutliche Unterschied zwischen der gesamtdeutschen Letalität und der unserer Kohorte ist somit insbesondere deshalb interessant, weil die Zahlen zeitgleich erhoben wurden, aus dem gleichen Gesundheitssystem stammen und somit oben genannte Einflussfaktoren ausgeschlossen werden können. Zudem ähnelt die von uns beschriebene Kohorte beatmeter Intensivpatienten im Hinblick auf Alter und Komorbiditäten in vielen Aspekten der gesamtdeutschen Kohorte [1]. Allerdings waren mehr als doppelt so viele Patienten adipös (29 % vs. 13 %), und mit 81 % war das männliche Geschlecht überrepräsentiert (deutschlandweit 66 %). Zwar wiesen weniger Patienten als Komorbidität eine chronische Niereninsuffizienz auf (14 % vs. 24 %), dennoch benötigten deutlich mehr Patienten während des intensivmedizinischen Aufenthalts im Rahmen eines akuten Nierenversagens eine Nierenersatztherapie (43 % vs. 27 %). Die Letalität der LMU-Kohorte lag mit 29 % jedoch weit unter der gesamtdeutschen Letalität. Doch wie lassen sich die Unterschiede in der Letalität erklären?

Diese Frage lässt sich anhand der vorliegenden Daten nicht wissenschaftlich beantworten. Eine geringere Erkrankungsschwere der LMU-Kohorte ist aufgrund des hohen SAPS II von 62 Punkten, der hohen Rate an akutem Nierenversagen und der Tatsache, dass über 27 % der Patienten von externen akutmedizinischen Krankenhäusern zur Therapieeskalation ins Zentrum zuverlegt wurden, nicht anzunehmen. Vergleichbare Scores zur Klassifikation der Erkrankungsschwere liegen in der deutschlandweiten Erhebung und in internationalen Vergleichskollektiven [4–8] nicht vor. Beachtet werden muss aber einerseits die möglicherweise unpräzise Letalitätsschätzung, bedingt durch den monozentrischen Studienansatz, und andererseits, dass unklar bleibt, bei wie vielen der Intensivpatienten in der gesamtdeutschen Kohorte die Therapie, z. B. bedingt durch den Patientenwillen, limitiert wurde. Insgesamt sind die deutschlandweit erhobenen multizentrischen Querschnittsdaten, die Meldungen aus Krankenhäusern aller Versorgungsstufen beinhalten, notwendigerweise nur eingeschränkt mit Daten aus einer monozentrischen Erhebung aus einem Universitätsklinikum vergleichbar.

Fernab der oben genannten offenen Fragen sollen im Folgenden mögliche Einflussfaktoren, die die Letalität der Münchner Kohorte positiv beeinflusst haben könnten, diskutiert werden.

1. Gabe von Steroiden bei COVID-19:
  Etwa 25 % der COVID-19-Patienten der LMU-Kohorte wurden nach Ausschluss einer Superinfektion prolongiert mit niedrigdosiertem Steroid behandelt. Dies geschah im Sinne einer „Rescue“-Therapie durch Erfahrung bei ARDS anderer Ätiologie und entgegen den zu diesem Zeitpunkt aktuellen nationalen und internationalen Empfehlungen, die initial in Europa vom Gebrauch von Steroiden bei COVID-19 abrieten [9, 10]. Die „Recovery“-Studie zum Einsatz von Steroiden bei COVID-19 wurde erst am 22.06.2020 in ihrer vorläufigen Version veröffentlicht [11]. Sie konnte eindrücklich eine reduzierte 28-Tage-Letalität bei schwer betroffenen COVID-19-Patienten zeigen, die Dexamethason (6 mg täglich für 10 Tage) erhielten. Wichtig sind die Identifikation geeigneter Patienten sowie eine strikte Infektionssurveillance.
  Bei 25 % unserer Patienten gelang während des intensivmedizinischen Verlaufes der Nachweis eines als relevant eingestuften weiteren Erregers aus respiratorischem Material. Die Datenlage zu Ko- bzw. Superinfektionen bei COVID-19 ist insgesamt schlecht [12], und systematische Analysen fehlen. Dennoch erscheint diese Rate vergleichsweise hoch. Ein möglicher Zusammenhang mit der immunmodulatorischen Therapie muss kritisch beobachtet werden.
  Die Gabe von Steroiden nach dem Schema der Recovery-Studie fand nun Eingang in die aktualisierten Empfehlungen der Fachgesellschaften [13]. Eine Reduktion der allgemeinen Letalitätszahlen durch eine entsprechende Therapieanpassung ist denkbar.
2. Spezifische antivirale Therapie:
  Wie flüchtig die medizinischen Informationen in der Frühphase der Pandemie waren, kann anhand der unterschiedlichen, spezifischen antiviralen Behandlungsregimes am LMU-Klinikum im Verlauf der ersten Welle eindrucksvoll nachvollzogen werden. Zu Beginn der Pandemie wurden im Rahmen individueller Heilversuche ohne bestehende Evidenz 9 verschiedene Medikamente verabreicht, deren Einsatz mit der Zunahme an hochwertigen Studien nicht mehr außerhalb von kontrollierten Studien gerechtfertigt schien. Dieser breite Einsatz von Medikamenten im Rahmen individueller Heilversuche auf dem Boden einer unfertigen Datenlage ist in der jüngeren Geschichte der deutschen Intensivmedizin einzigartig, in der Kriterien der evidenzbasierten Medizin als Grundlage therapeutischer Entscheidungen gelten. Insgesamt fällt auf, dass zu Beginn der Pandemie nahezu alle Patienten z. T. in Kombinationstherapie mehrere vermeintlich spezifische antivirale Substanzen erhalten haben. Durch zunehmende klinische Erfahrung und Studienergebnisse kamen im späteren Verlauf immer weniger Substanzen zum Einsatz.
3. Antikoagulation:
  Thrombembolische Ereignisse, auch Mikrothromben in der pulmonalen Strombahn, sind eine häufige Komplikation bei COVID-19, möglicherweise mitunter auch das pathophysiologische Korrelat der schweren Erkrankung [14]. Die COVID-19-assoziierte Koagulopathie sowie die pulmonale intravaskuläre Koagulopathie sind mittlerweile feststehende Begriffe [13]. Durch differenzierte Gerinnungsdiagnostik und deren Vergleich mit einer historischen Kohorte konnte das prokoagulatorische Potenzial der COVID-19-Patienten in den LMU-Kliniken bereits früh erkannt und beschrieben werden [15, 16]. Alle intensivpflichtigen COVID-19-Patienten wurden nach individueller Nutzen-Risiko-Kalkulation ab Beginn der Pandemie möglichst therapeutisch antikoaguliert und zwar bereits vor der Aufnahme dieser Empfehlung in die Algorithmen der Fachgesellschaften [9]. Nur 21 % der Münchner Kohorte hatten thrombembolische Komplikationen und damit deutlich weniger als in entsprechenden intensivmedizinischen Vergleichskollektiven mit Raten von 31–59 % [17, 18].
4. Beatmung:
  Unser klinikinterner Algorithmus sah vor, dass COVID-19-Patienten vor Intubation engmaschig auf Zeichen der drohenden respiratorischen Erschöpfung überwacht werden. Eine primäre NIV-Therapie erfolgt nur in speziellen Ausnahmesituationen. Dies ist insbesondere vor dem Hintergrund der Debatte interessant, ob COVID-19-Patienten zu früh intubiert werden und der nichtinvasiven Beatmung ein höherer Stellenwert eingeräumt werden sollte [19, 20]. Das Vorbehalten der NIV-Therapie ausschließlich für spezielle Ausnahmesituationen entspricht nicht mehr den aktuellen Empfehlungen [9]. Trotzdem war die Letalität in unserem Kollektiv mit klar definierten Intubationskriterien niedriger als in den genannten Vergleichskollektiven. Die Vergleichszahlen der gesamtdeutschen Kohorte zeigen eine mit 45 % niedrigere Letalität bei Patienten, die nur nichtinvasiv beatmet werden, im Vergleich zu den invasiv beatmeten Patienten [1]. Die Identifikation von für die NIV-Therapie geeigneten Patienten bleibt eine Herausforderung.

Zusammengefasst zeigen die genannten Beispiele, dass die fundierte Erfahrung mit dem schweren Lungenversagen, der kritische Umgang mit der wenig validen und sich rasch ändernden Datenlage, die interdisziplinäre Auseinandersetzung mit aufkommenden Fragestellungen sowie die rasche Implementierung an die eigene Klinikstruktur angepasster Behandlungsalgorithmen dazu beigetragen haben könnten, das Outcome der Patienten zu verbessern.

Einschränkend muss gesagt werden, dass wir nicht statistisch belegen können, welche dieser vorgestellten Maßnahmen dazu beigetragen haben könnte, die Letalität unserer Kohorte zu reduzieren, bzw. ob überhaupt irgendeine dieser Maßnahmen ursächlich damit korreliert. Herausgearbeitet wurden lediglich Aspekte, die aufgrund der Möglichkeiten einer universitären Organisationsstruktur mit Behandlungsschwerpunkt ARDS zu Unterschieden in der Behandlung geführt haben könnten.

Eine Aufarbeitung der deutschlandweiten Letalitätsdaten auch mit Prüfung von Zentrumseffekten wäre hilfreich.

Kritisch hinterfragt werden muss die hohe Letalität in der Subgruppe der ECMO-Patienten, die mit 86 % noch deutlich über der ohnehin hohen deutschlandweiten Letalität von 71 % liegt [1]. Die Letalität des schweren Non-COVID-19-ARDS mit ECMO-Therapie ist mit 35 % weitaus niedriger [21]. Von den insgesamt 10 an der ECMO behandelten Patienten befinden sich allerdings derzeit noch 3 in stationärer Behandlung und konnten daher nicht eingeschlossen werden. Eine Abnahme der Letalität ist somit perspektivisch möglich.

Das COVID-19-ARDS präsentiert sich als komplexe Multiorganerkrankung mit krankheitsspezifischen Besonderheiten, aber auch vielen Parallelen zum akuten Lungenversagen anderer Ätiologie. Analog zu den Empfehlungen der S3-Leitlinie zur invasiven Beatmung [22], sollte auch beim schweren COVID-19-ARDS die frühzeitige Verlegung in ein Zentrum erfolgen. Die Koordination ist über das DIVI-Intensivregister problemlos möglich. Die Transportrisiken sind gering [22]. Im Rahmen der ersten Welle waren jederzeit ausreichend Intensiv- und Transportkapazitäten jeder Versorgungsstufe vorhanden, obwohl die intensivmedizinischen Behandlungsverläufe in unserem Kollektiv mit im Median 18 Tagen langwierig waren. Im Falle einer Ressourcenknappheit mit Überlastung des Gesundheitssystems ermutigen wir zu einem engmaschigen, niederschwelligen Austausch zwischen den Kliniken der unterschiedlichen Versorgungsstufen zum Erfahrungs- und Wissenstransfer, aber auch zur gemeinsamen Identifikation von Patienten, die von einer Verlegung in ein ARDS-Zentrum profitieren könnten.

## Fazit für die Praxis

- Die intensivmedizinische Behandlung von COVID-19-Patienten ist langwierig, aber erfolgreich.
- Die Letalität von intensivpflichtigen COVID-19-Patienten unterscheidet sich auch in Deutschland erheblich.
- In Anbetracht von immer neuen Studienergebnissen befindet sich die optimale Behandlung von beatmeten COVID-19-Patienten weiterhin im Wandel.
- Schwerstkranke Patienten sollten nach Ansicht der Autoren in einem ARDS-Zentrum behandelt werden, solange ausreichende Ressourcen verfügbar sind.

## Abkürzungen

ARDS: „Acute respiratory distress syndrome“
BMI: Body-Mass-Index
COVID: „Coronavirus disease“
ECMO: Extrakorporale Membranoxygenierung
IQR: „Interquartile range“
LMU: Ludwig-Maximilians-Universität
NIV: Nichtinvasive Beatmung
PCR: Polymerase-Ketten-Reaktion
SAPS: „Simplified acute physiology score“
SARS-CoV‑2: „Severe acute respiratory syndrome coronavirus type 2“
